# The prognostic impact of specialist cardiology input in patients admitted for heart failure and normal ejection fraction

**DOI:** 10.1002/ehf2.14440

**Published:** 2023-06-25

**Authors:** Antonio Cannata, Layla Badawy, Anawinla Ta Anyu, Jack Samways, Mark Sweeney, Antonio Jordan‐Rios, Rosita Zakeri, Paul A. Scott, Susan Piper, Carla M. Plymen, Theresa A. McDonagh, Daniel I. Bromage

**Affiliations:** ^1^ Department of Cardiology King's College Hospital London London UK; ^2^ School of Cardiovascular Medicine and Sciences King's College London British Heart Foundation Centre of Excellence London UK; ^3^ Department of Cardiology, Hammersmith Hospital Imperial College Healthcare NHS Trust London UK

**Keywords:** Heart failure, Heart failure with normal ejection fraction, HFnEF, Mortality, Specialist input

## Abstract

**Aims:**

Specialist cardiology care is associated with a prognostic benefit in patients with heart failure (HF) with reduced ejection fraction (HFrEF) admitted with decompensated HF. However, up to one third of patients admitted with HF and normal ejection fraction (HFnEF) do not receive specialist cardiology input. Whether this has prognostic implications is unknown.

**Methods and results:**

Data on patients hospitalized with HFnEF from two tertiary centres were analysed. The primary outcome measure was all‐cause mortality during follow‐up. The secondary outcome was in‐hospital mortality. A total of 1413 patients were included in the study. Of these, 23% (*n* = 322) did not receive in‐hospital specialist cardiology input. Patients seen by a cardiologist were less likely to have hypertension (73% vs. 79%, *P* = 0.03) and respiratory co‐morbidities (25% vs. 31%, *P* = 0.02) compared with those who did not receive specialist input. Similarly, clinical presentation was more severe for those who received specialist input (New York Heart Association III/IV 83% vs. 75% respectively, *P* = 0.003; moderate‐to‐severe peripheral oedema 65% vs. 54%, *P* < 0.001). Medical management was similar, except for a higher use of diuretics (90% vs. 86%, *P* = 0.04) and a longer length of stay for patients who received specialist input (9 vs. 4 days, *P* < 0.001). Long‐term outcomes were comparable between patients who received specialist input and those who did not. However, specialist input was independently associated with lower in‐hospital mortality (hazard ratio 0.19, confidence interval 0.09–0.43, *P* < 0.001).

**Conclusions:**

In‐hospital cardiology specialist input has no long‐term prognostic advantage in patients with HFnEF but is independently associated with reduced in‐hospital mortality.

## Introduction

Patients with chronic heart failure (HF) are frequently admitted with episodes of acute decompensated HF. Specialist cardiology care is associated with prognostic benefit in patients with heart failure with reduced ejection fraction (HFrEF), which may relate to better uptake of evidence‐based interventions.^1^, ^2^, ^3^ However, patients admitted with HF and left ventricular ejection fractions (LVEF) of ≥50%, that is, HF with ‘normal’ ejection fraction (HFnEF), have a similar risk profile than those with HFrEF.^4^, ^5^, ^6^, ^7^, ^8^, ^9^, ^10^ At present, there is a paucity of guideline‐recommended therapies for this cohort of patients, limited in the recent ESC guidelines to diuretics for ‘preserved’ ejection fraction (HFpEF) and valve intervention in a subset of patients with valvular heart disease.^11^ To date, it is unknown whether the prognostic benefit of specialist cardiology input applies to those patients with HFnEF. The aim of the present study was to describe the association of specialist cardiology input and outcomes in patients admitted with decompensated heart failure and normal LVEF.

## Methods

### Study design, population, and definitions

This was a prospective cohort study of consecutive, unselected patients hospitalized with signs and symptoms of HF and LVEF ≥50%,^4^, ^5^, ^6^, ^7^, ^11^ between March 2012 and November 2020. Patients older than 18 years at the time of admission and hospitalized in two tertiary referral centres in London, UK (King's College Hospital and Imperial College Healthcare NHS Trust, comprising Hammersmith, St Mary's and Charing Cross Hospitals) were included. Data were collected as part of the National Heart Failure Audit (NHFA) for England and Wales, which collects data on acute HF hospitalizations from NHS Trusts in England and Health Boards in Wales. Patients were entered into the audit if they had a primary discharge diagnosis of acute HF based on the following ICD codes (I11.0 Hypertensive heart disease with (congestive) heart failure; I25.5 Ischaemic cardiomyopathy; I42.0 Dilated cardiomyopathy; I42.9 Cardiomyopathy, unspecified; I50.0 Congestive heart failure; I50.1 Left ventricular failure; I50.9 Heart failure, unspecified.

Patients were classified whether they have received in‐hospital cardiology specialist input or not. Specialist input was defined as the referral and subsequent consultation with either a cardiologist or an HF specialist during the admission. Valve heart disease was defined as a moderate or severe valve lesion by the reporting sonographer using British Society of Echocardiography definitions.^12^ *Table* *S1* summarizes the ‘Strengthening the Reporting of Observational Studies in Epidemiology’ checklist for reporting on observational research.^13^, ^14^

### Baseline characteristics

For each patient, clinician‐collected data on demographics, co‐morbidities and cardiovascular risk factors (mandatory fields in the NHFA), and variables previously shown to be associated with outcomes^15^ were recorded, including age, sex, creatinine, systolic blood pressure, and history of cardiovascular disease. The standard dataset used for the NHFA is available from NICOR (https://www.nicor.org.uk/national‐cardiac‐audit‐programme/datasets/).

### Outcome measures

The primary outcome of the study was all‐cause mortality during follow‐up. Mortality status was identified from NHS Digital using the patient NHS number, a 10‐digit unique identifier assigned at first interaction with the healthcare system, date of birth, patient postcode and sex. The secondary outcome was in‐hospital mortality collected from hospital records.

### Statistical analysis

Variables are expressed as mean and standard deviation, median and interquartile range, or counts and percentage, as appropriate. Baseline characteristics were compared using the Pearson's *χ* ^2^ test for categorical variables or the analysis of variance for continuous variables or by the non‐parametric Mann–Whitney *U* test when necessary. Normality of distribution was assessed using the Shapiro–Wilk's test. Survival curves for in‐hospital mortality were estimated and compared between years by means of the log‐rank test. To investigate the impact of specialist input, cause‐specific univariable and multivariable Cox regression models were estimated from an unselected list of candidate prognostic variables obtained from the univariable analyses (i.e. those with a *P*‐value ≤0.1). A sensitivity analysis was performed to assess the impact of admitting hospital. The IBM‐SPSS statistical software version 25.0 (IBM Corp., NY, USA) and the software R (R Foundation for Statistical Computing, Vienna, Austria. URL https://www.r‐project.org/) were used for statistical analyses.

## Results

### Study population

A total of 1413 patients with HFnEF were included. Of these, approximately one quarter (*n* = 322, 23%) did not receive speciality cardiology input during their hospitalization. Baseline characteristics are shown in *Table* *1*. Compared with those who did not receive specialist input, patients seen by a cardiologist were younger (79 vs. 80 years, *P* = 0.03), less likely to have hypertension (73% vs. 79%, *P* = 0.03) and respiratory co‐morbidities (25% vs. 31%, *P* = 0.02). Clinical presentation was more severe in patients who received specialist input compared with those not seen by a cardiologist in‐hospital (New York Heart Association III/IV at admission in 83% vs. 75%, *P* = 0.003, and moderate to severe peripheral oedema in 65% vs. 54%, *P* < 0.001). Both cohorts received similar medical management, except for a higher use of diuretics in patients seen by a cardiologist (90% vs. 86%, *P* = 0.04). Median length of stay was significantly longer for patients received specialist input compared with those who did not (9 vs. 4 days, *P* < 0.001). Weight change was greater in patients seen by a specialist compared with those without specialist input (−4.1 ± 5.1 kg vs. −1.7 ± 2.9 kg, *P* < 0.01). Overall, a higher percentage of patients who received cardiology input had a subsequent scheduled cardiology follow‐up (62% for those seen by cardiology vs. 28% for those not seen, *P* < 0.001).

**Table 1:** Baseline characteristics of the population

	No. of specialist input	Specialist input	*P*‐value
Number of patients	322	1091	
Age, years	80 [71; 87]	79 [70; 86]	**0.03**
Males, *n* (%)	120 (37%)	457 (42%)	0.14
Ethnicity			
White, *n* (%)	131 (54%)	400 (48%)	0.18
Black, *n* (%)	74 (30%)	255 (31%)	
Other races, *n* (%)	39 (16%)	173 (21%)	
Change in body weight, kg	1.7 ± 2.8	4.1 ± 5.1	**<0.001**
Heart rate at admission, b.p.m.	78 [64;91]	79 [67;92]	0.12
Sinus rhythm, *n* (%)	133 (49%)	388 (44%)	0.16
Atrial fibrillation, *n* (%)	139 (51%)	493 (56%)	
Systolic blood pressure, mmHg	138 [120;155]	136 [118;159]	0.98
NYHA class III/IV, *n* (%)	230 (75%)	891 (83%)	**0.003**
Moderate–severe oedema, *n* (%)	166 (54%)	692 (65%)	**<0.001**
Co‐morbidities			
IHD, *n* (%)	90 (30%)	353 (34%)	0.29
Pre‐existing valve disease, *n* (%)	106 (34%)	522 (49%)	**<0.001**
Hypertension, *n* (%)	249 (79%)	782 (73%)	**0.03**
Diabetes, *n* (%)	141 (44%)	498 (46%)	0.59
Respiratory disease, *n* (%)	102 (31%)	274 (25%)	**0.02**
Creatinine, μmol/L	104 [77;148]	109 [81;158]	0.21
Potassium, mEq/L	4.2 [3.8;4.6]	4.2 [3.9;4.6]	0.29
Cardiology follow‐up, *n* (%)	84 (28%)	633 (63%)	**<0.001**
Discharge medications			
ACEi/ARBs, *n* (%)	129 (43%)	478 (47%)	0.43
Beta‐blocker, *n* (%)	163 (53%)	588 (57%)	0.66
Diuretics, *n* (%)	266 (86%)	943 (90%)	**0.04**
MRAs, *n* (%)	34 (11%)	262 (25%)	**<0.001**
Length of stay, days	4 [2;9]		
In‐hospital mortality, *n* (%)	13 (4%)	23 (2%)	**<0.001**
All‐cause mortality, *n* (%)	110 (54%)	221 (54%)	0.083

*Note*: Values in bold indicate *P* < 0.05.

ACEi, angiotensin converting enzyme inhibitors; ARBs, angiotensin receptor blockers; IHD, ischaemic heart disease; MRA, mineralocorticoid receptor antagonist; NYHA, New York Heart Association.

### Outcomes

**Figure 1:**
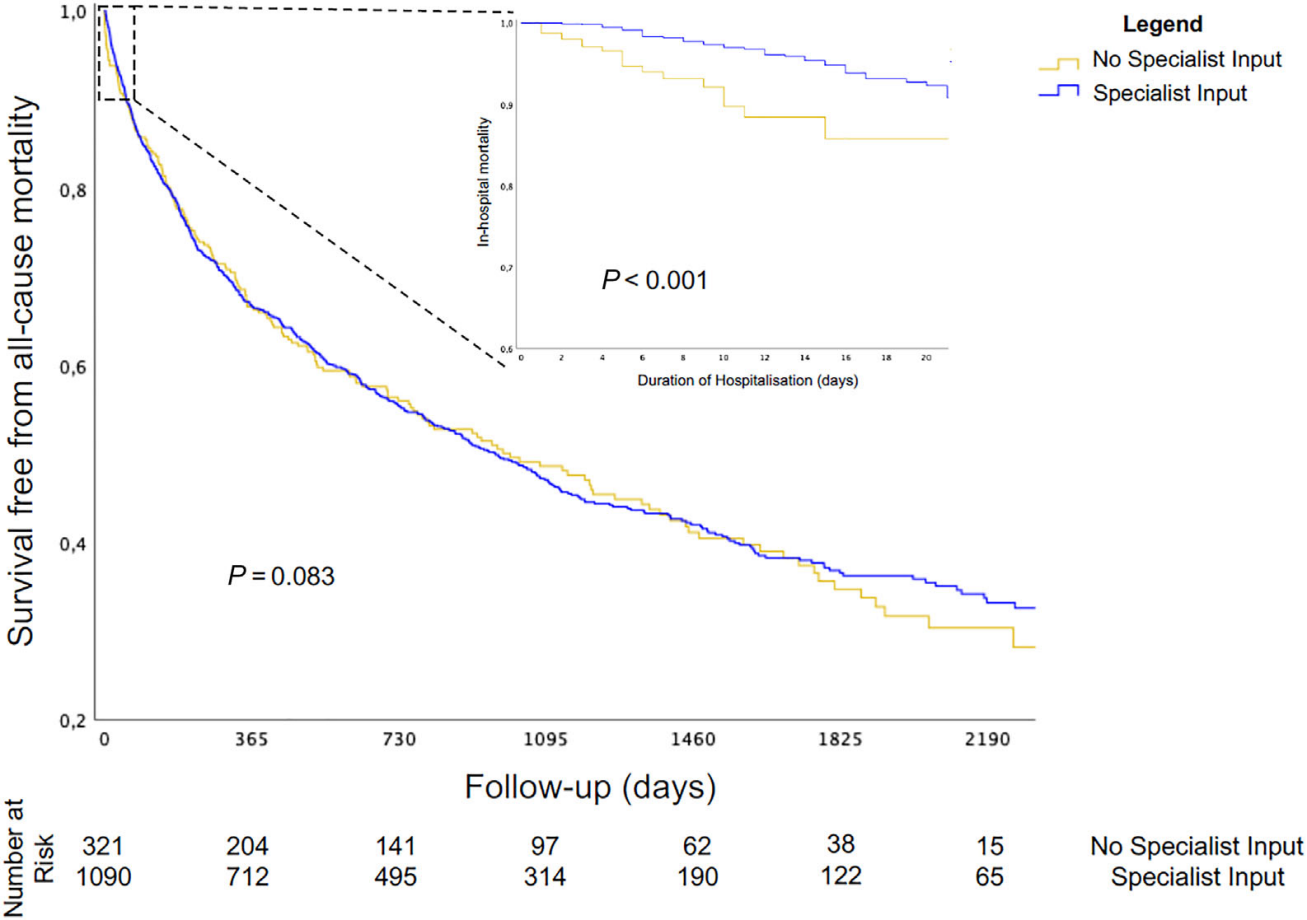
Kaplan–Meier curves showing survival free from the primary outcome (main panel) of all‐cause mortality in patients with and without specialist input. Inset shows the secondary outcome of in‐hospital mortality.

**Figure 2:**
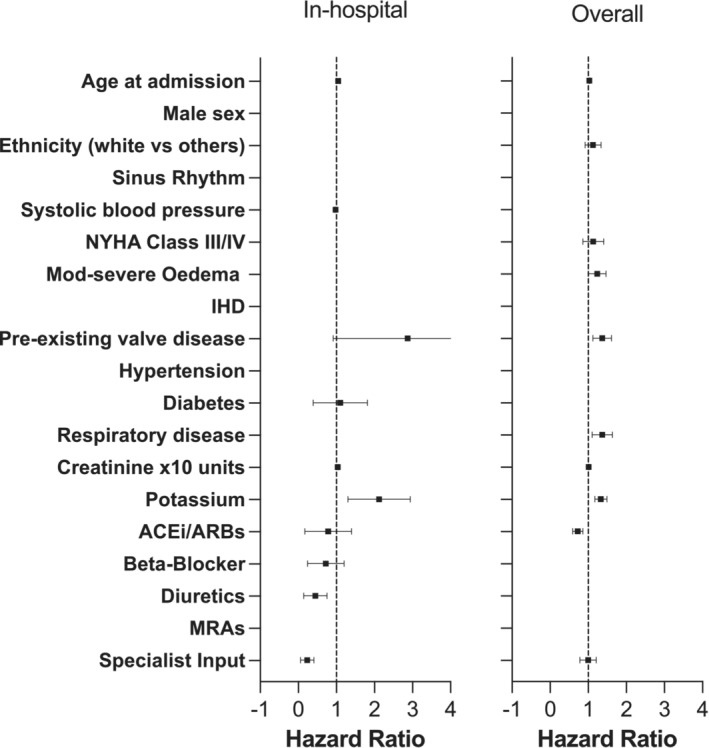
Forest plot for the adjusted risk of in‐hospital (left panel) and overall (right panel) mortality.

Over a median follow‐up of 20 [interquartile range 7–39] months, 788 patients died (56%). There was no significant difference in the primary outcome between patients who received specialist input and those who did not (*P* = 0.83; *Figure* *1*). After adjustment for baseline characteristics and co‐morbidities, there was no association between specialist input and all‐cause mortality during follow‐up (hazard ratio 0.98, 95% confidence interval 0.79–1.22, *P* = 0.88; *Table* *2*, *Figure* *2*).

**Table 2:** Univariate and multivariate Cox proportional hazard model for all‐cause mortality

	Univariate				Multivariate			
	HR	95% CI		*P*‐value	HR		95% CI	*P*‐value
Age	1.02	1.02	1.03	**<0.001**	1.03	1.02	1.04	**<0.001**
Male sex	1.03	0.89	1.18	0.71				
Ethnicity (White vs. others)	1.24	1.05	1.46	**0.009**	1.11	0.92	1.34	0.26
Sinus rhythm	1.13	0.97	1.33	0.11				
Systolic blood pressure	0.99	0.99	1.01	0.29				
NYHA class III/IV	1.23	1.04	1.47	**0.04**	1.11	0.87	1.41	0.39
Mod‐severe oedema	1.22	1.05	1.41	**0.009**	1.22	1.01	1.47	**0.04**
IHD	1.12	0.96	1.31	0.13				
Pre‐existing valve disease	1.34	1.17	1.55	**<0.001**	1.35	1.13	1.62	**0.01**
Hypertension	0.89	0.76	1.04	0.15				
Diabetes	1.03	0.89	1.19	0.67				
Respiratory disease	1.23	1.05	1.43	**0.009**	1.35	1.11	1.64	**0.002**
Creatinine ×10 units	1.01	1.00	1.01	**0.003**	1.01	1.00	1.02	**<0.001**
Potassium	1.31	1.19	1.44	**<0.001**	1.33	1.17	1.49	**<0.001**
ACEi/ARBs	0.66	0.57	0.77	**<0.001**	0.72	0.59	0.86	**<0.001**
Beta‐blocker	0.88	0.77	1.03	0.11				
Diuretics	0.93	0.74	1.17	0.54				
MRAs	0.89	0.75	1.07	0.23				
Specialist input	0.98	0.83	1.16	0.81	0.98	0.79	1.22	0.88

*Note*: Values in bold indicate *P* < 0.05.

ACEi, angiotensin converting enzyme inhibitors; ARBs, angiotensin receptor blockers; CI, confidence interval; HR, hazard ratio; IHD, ischaemic heart disease; MRA, mineralocorticoid receptor antagonist; NYHA, New York Heart Association.

However, in the hospital setting, 88 patients (6%) died during their index admission. Specialist cardiology input was associated with lower in‐hospital mortality (*P* < 0.001; *Figure* *1*). At multivariable analysis, specialist input remained an independent predictor of hospital survival in patients with HFnEF (hazard ratio 0.19, confidence interval 0.09–0.43, *P* < 0.001; *Table* *3*, *Figure* *2*). When an interaction term was added to the model to investigate the role of specialist cardiology input in each co‐morbidity, no significant interaction between specialist input and any co‐morbidity was found, with similar predictive values between models. Results were consistent between admitting hospitals with respect to the primary and secondary outcomes.

**Table 3:** Univariate and Multivariate Cox‐proportional Hazard model for in‐hospital mortality

	Univariate				Multivariate			
Variable	HR	95% CI		*P*‐value	HR	95% CI		*P*‐value
Age at admission	1.04	1.02	1.06	**<0.001**	1.05	**0.007**	1.09	
Male sex	1.02	0.66	1.57	0.93				
Ethnicity (White vs. others)	0.92	0.58	1.44	0.72				
Sinus rhythm	0.91	0.55	1.47	0.68				
Systolic blood pressure	0.96	0.94	0.99	**0.003**	0.98	0.96	0.99	**0.002**
NYHA Class III/IV, *n* (%)	0.66	0.34	1.26	0.22				
Mod‐severe oedema	1.13	0.72	1.79	0.59				
IHD	0.81	0.52	1.27	0.36				
Pre‐existing valve disease	2.03	1.30	3.18	**0.002**	2.43	1.17	5.01	**0.02**
Hypertension	0.87	0.56	1.37	0.55				
Diabetes	0.63	0.41	0.97	**0.04**	0.95	0.48	1.88	0.87
Respiratory disease	0.66	0.39	1.11	0.12				
Creatinine ×10 units	1.03	1.01	1.04	**<0.001**	1.03	1.01	1.06	**0.008**
Potassium	2.14	1.72	2.65	**<0.001**	2.02	1.36	2.99	**<0.001**
ACEi/ARBs	0.34	0.15	0.77	**0.01**	0.62	0.27	1.46	0.28
Beta‐Blocker	0.51	0.26	0.97	**0.04**	0.61	0.31	1.25	0.18
Diuretics	0.23	0.12	0.44	**<0.001**	0.38	0.18	0.78	**0.009**
MRAs	0.56	0.25	1.27	0.16				
Specialist Input	0.40	0.24	0.66	**<0.001**	0.19	0.09	0.43	**<0.001**

*Note*: Values in bold indicate *P* < 0.05.

ACEi, angiotensin converting enzyme inhibitors; ARBs, angiotensin receptor blockers; CI, confidence interval; HR, hazard ratio; IHD, ischaemic heart disease; MRA, mineralocorticoid receptor antagonist; NYHA, New York Heart Association.

## Discussion

HF is a complex clinical syndrome that is frequently associated with a median of five co‐morbid conditions.^10^, ^11^, ^16^, ^17^ Hospitalizations are common in HF patients, either caused by *de novo* HF or decompensation of pre‐existing chronic HF. Such decompensation may relate to one or more exacerbating conditions, such as an acute coronary syndrome, arrhythmia, non‐adherence to medication, or intercurrent infection, which further adds to the complexity of the disease.

In the United Kingdom, patients hospitalized with HF may be managed principally by specialist cardiology teams or general physicians, and management is often shared and inter‐disciplinary. However, hospital pathways, the number of patients with confirmed or suspected HF, and the availability of specialist cardiology multi‐disciplinary teams vary, such that not all patients hospitalized with HF receive specialist cardiology input during their admission. In the United Kingdom, approximately 40% of patients admitted with HF are managed in cardiology wards while the rest are mainly managed in general medical wards or by geriatricians.^18^ Therefore, given the larger number of patients managed in general medical wards, specialist cardiology input has an important role in the optimization of care for these patients. Indeed, specialist cardiology care helps facilitate both the initiation and up‐titration guideline‐directed medical treatment for HFrEF and referral for invasive treatment, where necessary. As a result, specialist cardiology input is recommended for patients admitted with HF as it is associated with improved prognosis.^11^ The main beneficial effect of specialist cardiology input is for HFrEF, where guideline‐directed medical therapy is able to change the trajectory of the disease. Conversely, no studies have specifically examined whether cardiology input in patients with normal LVEF is associated with improvement in either in‐hospital or long‐term survival.

In our analysis, we identified 1413 patients who were hospitalized with HFnEF, of whom approximately three quarters were seen by a cardiology specialist. Approximately one quarter of patients with a diagnosis of HFnEF were either not referred to cardiology or were discharged or died before receiving specialist input.

Patients seen by cardiology during their admission were slightly younger, but had more severe signs and symptoms of HF. The different clinical profile may have prompted a more intensive diuretic treatment, which, combined with a longer length of stay, led to a greater weight change during admission. These results are consistent with the latest report from the NHFA where patients with HF receiving specialist care have 4 days longer admission compared with those not seen by cardiologist.^18^ Furthermore, the different demographic and co‐morbidity profiles of patients seen by cardiologist may reflect the referral of more severe cases, a different rate of optimization of medical and device therapy and/or a greater priority given to achieving clinical stability before discharge.

Approximately half of patients were discharged on renin‐angiotensin‐aldosterone system inhibitors or beta‐blockers, which was similar between groups except for mineralocorticoid receptor antagonists, which were more frequently prescribed in patients seen by a specialist.

In our analysis, long‐term survival in patients with HFnEF who received in‐hospital specialist cardiology input was comparable to those who did not receive specialist input. After adjustment, respiratory co‐morbidities and pre‐existing moderate–severe valve disease were independently associated with worse prognosis, together with older age, worse congestion status, chronic kidney disease, and hyperkalaemia. The prognostic role of specialist cardiology input in an area in which large randomized clinical trials have, until recently, failed to demonstrate a clear prognostic benefit of available medical or device treatment is debatable. The comparable long‐term event rate between HFnEF patients who received in‐hospital specialist cardiology input and those who did not may relate to the paucity of guideline‐recommended treatments in this cohort of patients. To date, only diuretic therapy has a class I recommendation in the current guidelines for patients with HFpEF.^11^ Recent analyses suggest potential benefit in reducing hospitalization with neurohormonal modulating therapies and/or sodium‐glucose co‐transporter 2 inhibitors (SGLT2) in the LVEF range included in this study.^19^, ^20^, ^21^ However, despite the evidence generated by the EMPEROR‐Preserved and the DELIVER trials using SGLT2i in patients with HFpEF, the use of SGLT2i is not yet approved by regulatory agencies in some countries or yet recommended in the guidelines for the treatment of HFpEF.

The widespread uptake of emerging evidence‐based medication may alter the association of specialist input with outcome in patients with HFnEF, and ongoing research will define the HFnEF phenotypes that may achieve a prognostic benefit from either specific cardiology or other specialist care (respiratory, for example).^16^

Interestingly, inpatient specialist cardiology input was associated with significantly lower in‐hospital mortality in HFnEF patients. After adjustment, specialist cardiology input persisted as an independent prognostic predictor together with older age, admission systolic blood pressure, pre‐existing valve disease, creatinine, potassium, and diuretic therapy. Receiving inpatient specialist cardiology input was strongly associated with higher survival. The reasons for this are largely unknown but may relate to more aggressive decongestion in patients seen by a HF specialist during hospitalization.

To our knowledge, this is the first study to investigate the association of in‐hospital specialist input and outcome in HFnEF. The results of EMPEROR‐Preserved^20^ and DELIVER^22^ introduced novel agents that are effective in patients with normal LVEF. However, both trials demonstrated a benefit largely in reducing hospitalizations, rather than cardiovascular mortality, highlighting the need for a better pathophysiological understanding of the disease. This may necessitate a more important role for specialist cardiology care in these patients. However, so far, specialist management of patients with normal LVEF may not always be required and further studies are warranted to investigate the role of specialist cardiology care in this setting.

### Limitations

This registry‐based analysis enrolled consecutive patients with confirmed HF from two large HF referral centre in London. Therefore, the results might not be generalizable to other places with different HFnEF incidence and require further confirmation in widespread multicentre analysis. Unfortunately, complete clinical data was not available for all patients. Patients included in this study had normal systolic function at echocardiogram defined as LVEF ≥50%. HFA‐PEFF and HF2‐PEF scores are not routinely calculated in hospitalized patients. Therefore, we were unable to confirm the diagnosis of HFpEF using either a score,^17^, ^23^ invasive measurements, or the pragmatic approach recommended in the ESC guidelines.^11^ Furthermore, phenotyping valve disease and the details of any valve interventions are not known for all patients. Similarly, previous history of HF or hospitalizations were not routinely collected. Dosage of medications was not available for all patients. Although we performed an unselected approach for the variables of interest, potential unknown or unmeasured confounding cannot be entirely excluded. The analysis was not limited to only those with complete information to avoid introducing selection bias.

## Conclusions

In‐hospital cardiology specialist input is associated with comparable long‐term outcomes for patients with HFnEF compared with those who do not receive specialist input but is associated with reduced in‐hospital mortality. Further studies are necessary to investigate the subgroup of patients who may benefit from specialist cardiology input.

## Conflict of interest

The authors declare that they have no competing interests.

## Funding

AC is funded by a British Heart Foundation Clinical Research Training Fellowship (FS/CRTF/21/24175). DB is supported by the King's BHF Centre of Research Excellence (RE/18/2/34213).

## Supporting information


**Data S1.** Supporting Information.
